# Rates, causes and predictors of all-cause and avoidable mortality in 514 878 adults with and without intellectual disabilities in Scotland: a record linkage national cohort study

**DOI:** 10.1136/bmjopen-2024-089962

**Published:** 2025-02-12

**Authors:** Ewelina Rydzewska, Dewy Nijhof, Laura Hughes, Craig Melville, Michael Fleming, Daniel Mackay, Filip Sosenko, Laura Ward, Kirsty Dunn, Maria Truesdale, Deborah Cairns, Jill P. Pell, Grant M. A. Wyper, Bhautesh D. Jani, Fiona Barlow, Angela Henderson, Ruth Callander, Sally-Ann Cooper

**Affiliations:** 1 School of Health in Social Science, University of Edinburgh, Edinburgh, Scotland, UK; 2 School of Health and Wellbeing, University of Glasgow, Glasgow, Scotland, UK; 3 School of Medicine, University of Dundee, Dundee, Scotland, UK; 4 Population Health and Wellbeing, Public Health Scotland, Edinburgh, Scotland, UK; 5 Scottish Commission for People with Learning Disabilities, Glasgow, Scotland, UK

**Keywords:** Mortality, Public Health, Primary Care

## Abstract

**Abstract:**

**Background:**

Studies on avoidable mortality in adults with intellectual disabilities are limited, as are studies on causes of death.

**Objectives:**

We aimed to quantify mortality rates, and causes, and identify factors (i.e., age, sex, Scottish Index of Multiple Deprivation (SIMD)) related to avoidable mortality in adults with intellectual disabilities.

**Design:**

A record linkage national cohort study.

**Setting:**

A cohort of adults with intellectual disabilities with or without co-occurring autism, aged 25+ years and a randomly selected comparison group aged 25+ years without intellectual disabilities or autism identified from Scotland’s Census, 2011. Census records were linked to the National Records of Scotland Statutory Register of Deaths database to ascertain all deaths from 2011 to 2019.

**Participants:**

We analysed data on 14 477 adults with intellectual disabilities aged 25+ years and a randomly selected comparison group of 506 207 adults aged 25+ without intellectual disabilities identified from Scotland’s Census 2011.

**Primary and secondary outcome measures:**

We ran χ^2^ tests and t-tests to investigate individual characteristics and differences in age at death for adults with intellectual disabilities compared with peers in the general population. Cox proportional hazard models were fitted to calculate risk of mortality (all-cause, avoidable, treatable, preventable) unadjusted and adjusted for age, sex and SIMD. We then calculated mortality rates, using crude and indirect standardisation methods.

**Results:**

During the 8.5-year follow-up, 23.7% (crude death rate of 3033.3 per 100 000) of adults with intellectual disabilities died compared with 13.8% of controls. The median age at death among adults aged 25+ with intellectual disabilities was 65.0 years compared with 80.0 years for adults without intellectual disabilities. For all-cause mortality, the age-standardised mortality ratio (SMR) in the population with intellectual disabilities was 3.1 (95% CI 3.0 to 3.2). The SMRs were higher for the youngest age groups, women and in the most affluent areas. This was also the case for SMRs for avoidable, treatable and preventable deaths. For the population of adults with intellectual disabilities, 31.7% of recorded deaths were considered avoidable, 21.1% were treatable and 19.9% were preventable. In the controls, 18.2% of deaths were considered avoidable, 8.8% treatable and 14.7% preventable. Down syndrome and dementia were the two most commonly recorded underlying causes of death for people with intellectual disabilities while malignant neoplasm of bronchus and lung and acute myocardial infarction were most commonly recorded in the general population.

**Conclusions:**

Risk of all-cause, avoidable, treatable and preventable mortality was higher for adults with intellectual disabilities than their peers. The highest SMRs were observed for youngest adults, women and individuals living in the most affluent areas.

**STRENGTHS AND LIMITATIONS OF THIS STUDY**

- Unique study of avoidable mortality in adults with intellectual disabilities in a whole country population.
- High response rate of 94% and systematic enquiry of everyone regarding intellectual disabilities.
- Results of the study are generalisable to other adult populations in high-income countries.
- The records of death were taken from death certificates and not verified at postmortem.

## Introduction

 On average, people with intellectual disabilities have been reported to die 20 years younger than those without intellectual disabilities, including dying from causes considered to have been avoidable.^1^ In recent years, there have been several studies of deaths in adults with intellectual disabilities, that have attempted to reduce the limitations of previous studies such as small sample sizes, or non-representative populations. These studies have typically used record-linkage methods and reported standardised mortality ratios (SMRs) in the region of 2–4, higher in women than men, and at younger ages, though some report SMR to be only slightly above 1.^1^ Direct comparisons between studies are difficult due to differences in methods, reporting and ages studied; a tabulated overview is provided in Cooper *et al*.^2^

There is much less information on the most common underlying causes of death in adults with intellectual disabilities and less consistency in how these are reported (e.g., grouping by International Classification of Diseases, Tenth Revision (ICD-10) categories, or by individual causes of death) which limits comparisons between studies. One study reported the most common causes of death to be pneumonia, other respiratory diseases and diseases of the nervous system.^3^ Another reported diseases of the circulatory and respiratory systems to be the most common cause of death.^4^ A third reported that mortality rates due to influenza and pneumonia, septicaemia and aspiration pneumonia substantially exceeded the adult mortality rates in the general population.^5^ A fourth reported diseases of the circulatory system, neoplasm and the nervous system to be the most common cause of death.^6^ A fifth noted that people with intellectual disabilities had increased odds of presentation, admission or death from conditions defined as ambulatory care sensitive, which are potentially preventable, specifically vaccine-preventable respiratory disease, asthma, cellulitis and convulsions and epilepsy.^7^ Three studies reported cause-specific SMRs to be higher across most groups of disorders than in the general population.^2^,^4^

Avoidable mortality has also been little studied in adults with intellectual disabilities. Its definition includes preventable mortality (deaths which are preventable through public health interventions, e.g., deaths from infectious diseases that can be prevented by vaccination, or alcohol or drug-related deaths) and treatable (previously known as ‘amenable’) mortality (deaths amenable to timely and effective healthcare, e.g., deaths due to epilepsy, diabetes, or respiratory infections) while some causes of death can be both preventable and treatable.^8 9^ Recent studies, which have reported on avoidable deaths, suggest that up to 40% of deaths of adults with intellectual disabilities may be avoidable, compared with 28% of deaths in the general adult population.^2 4 10 11^

Specifically, one study, in which 961 adults aged 16–83 years with intellectual disabilities had clinical examinations in 2001–2004, found that 102 (38.9%) of the 262 deaths were avoidable, 78 (29.8%) were treatable and 51 (19.5%) were preventable, while 27 (10.3%) were classed as both treatable and preventable.^2^ In a study of 16 666 adults with intellectual disabilities from 343 general practices in the UK, 37.0% of all deaths among adults with intellectual disabilities were classified as being treatable, compared with 22.5% in the general population (HR 5.9; 95% CI 5.1 to 6.8).^4^ A study of 732 deaths in 19 362 adults aged 20+ years registered for intellectual disability services from 2005 to 2011 in New South Wales found that 31% of deaths were avoidable; higher than in the general population (17%).^6^ Two further studies reported data on children, young people and adults combined.^10 11^ Heslop *et al*^10^ undertook a population-based confidential inquiry of the deaths of 247 people with intellectual disabilities aged 4 years and older in southwest England who died between 1 June 2010 and 31 May 2012. Treatable deaths were more common in people with intellectual disabilities (37%) than in the general population (13%).^10^ Glover *et al*^11^ used general practice data for people with and without intellectual disabilities of all ages and reported that 44.7% of deaths were avoidable, with a higher proportion of deaths from causes classified as treatable, but a lower proportion from preventable causes compared with people without intellectual disabilities (actual figures not reported).^11^ Two studies investigated patterns of mortality in adults with intellectual and developmental disabilities from Ontario, Canada. One of them reported a higher all-cause (6.1% vs 1.6%) and amenable (21.4% vs 14.1%) mortality levels compared with the general population, but rates for all avoidable mortality were not provided.^12^ The other study reported 1 year age-standardised mortality rates for years 2011–2014 to be between 30.3 and 37.4 for Manitoba adults with intellectual and developmental disabilities compared with the matched comparison group, meaning that 30.3–37.4 times more deaths occurred in this population than would be expected to occur in the Ontario population.^13^ A further study on adults with intellectual and developmental disabilities from Manitoba, Canada reported crude avoidable premature mortality rates per 1000 person-years to be between 2.3 and 3.3 for years 2013–2015, meaning that avoidable premature mortality was 2.3–3.3 times more prevalent among Manitoba adults with intellectual and developmental disabilities compared with the matched comparison group.^14^ These studies demonstrated that rates of avoidable mortality are high in adults with intellectual disabilities, and higher than in the general population, suggesting that pervasive health inequalities may be contributing and that further investigation is necessary.

The aim of this study was to investigate deaths in adults with intellectual disabilities, compared with controls, for an entire country’s population from 2011 to 2019. For adults with intellectual disabilities compared with other adults, we investigated (a) age, sex and neighbourhood deprivation SMRs, (b) underlying cause of death, and all contributing factors in death by ICD-10 chapters, and most common specific causes and (c) the proportion of deaths considered avoidable, treatable and preventable.

## Methodology

### Patient and public involvement

This study was undertaken by the Scottish Learning Disabilities Observatory at the University of Glasgow due to the growing concern expressed by people with intellectual disabilities and their families about mortality. The Scottish Learning Disabilities Observatory’s steering group includes people with intellectual disabilities and partners from third-sector organisations. Results from this study will be disseminated to people with intellectual disabilities and their families in an easy-to-read version via the Scottish Learning Disabilities Observatory website and newsletters and in collaboration with the Scottish Commission for People with Learning Disabilities.

### Study sample, setting and process

94% of the Scottish population completed Scotland’s Census, 2011. We used these data to create a cohort of adults with intellectual disabilities with and without co-occurring autism, aged 25+ years at the census date (27 March 2011), and a randomly selected comparison group aged 25+ years without intellectual disabilities or autism from a 15% unmatched sample of the Scottish population also identified from Scotland’s Census, 2011. Their records were linked to the National Records of Scotland Statutory Register of Deaths database to ascertain all deaths up to 31 December 2019. Access to the anonymised linked data was given to the approved members of the research team via Scotland’s National Safe Haven. Full details on Scotland’s Census 2011 are available at: https://www.scotlandscensus.gov.uk/about/2011-census/. Further information on the record linkage and the cohorts has previously been reported in detail.^15^

### Variables

#### Intellectual disabilities

Scotland’s Census 2011 provides information on the number and characteristics of Scotland’s population and households on the Census day, 27 March 2011. The census is undertaken every 10 years. It includes people living in communal establishments (such as care homes and student halls of residence) as well as people living in private households. In 2011, the census in Scotland was estimated to have achieved a 94% response rate.^16^ The census team required the form to be completed by the head of household or joint head of household on behalf of all occupants in private households, and by the manager on behalf of all occupants in communal dwellings. It was a legal requirement to complete the census, and non-compliance or supplying false information could result in a fine of £1000. The census team followed up non-responders and also provided help in responding when it was needed; hence, the high 94% completion rate.

Scotland’s Census is probably one of few country censuses which identifies people with intellectual disabilities and distinguishes intellectual disabilities from specific learning disabilities such as dyslexia; indeed, it may be unique in this regard. Self-reporting/proxy reporting was used to identify people with intellectual disabilities from the Census questionnaire, question 20: ‘Do you have any of the following conditions which have lasted, or are expected to last, at least 12 months? Tick all that apply’. Respondents were given a choice of 10 response options, with option number (3) ‘learning disability (e.g., Down’s syndrome)’ being synonymous in the UK with the international term ‘intellectual disabilities’. The remaining response options were as follows: (1) deafness or partial hearing loss, (2) blindness or partial sight loss, (4) learning difficulty (e.g., dyslexia), (5) developmental disorder (e.g., autistic spectrum disorder or Asperger’s syndrome), (6) physical disability, (7) mental health condition, (8) long-term illness, disease or condition, (9) other condition and (10) no condition. Importantly, the question distinguished between intellectual disability (for which the term ‘learning disability’ is commonly used in the UK), learning difficulty (which in the UK is synonymous with the international term ‘specific learning disability’ such as dyslexia) and autism.

#### Age

Age was grouped into six categories: (1) 25–34 years, (2) 35–44 years, (3) 45–54 years, (4) 55–64 years, (5) 65–74 and (6) 75+ years, based on the census data.

#### Sex

Sex was coded into two categories of male and female, based on the census data.

#### Scottish Index of Multiple Deprivation (SIMD)

SIMD was grouped in population quintiles where SIMD 1 included the most deprived neighbourhoods and SIMD 5 corresponded with the most affluent neighbourhoods. SIMD was identified from postcodes, based on the census data, and calculated at datazone level.

#### Deaths

We used data from death certificates registered at National Records of Scotland, to identify the date of deaths, and underlying causes and all contributing factors in deaths for adults with intellectual disabilities and the general population comparison group. For the cause of death analyses, we analysed the underlying cause of death, defined as the disease or injury which initiated the chain of morbid events leading directly to death, or the accident/act which produced the fatal injury.^17^ We also analysed a broader composite outcome of all contributing factors in death, defined as a cause listed as either the underlying cause, secondary cause or a contributing factor. We used the Tenth Revision of the International Statistical Classification of Diseases and Related Health Problems (ICD-10)^18^ to group causes of death into categories. The cancer codes included C00.0–D48.9 inclusive. The analyses were restricted to deaths recorded between 28 March 2011 and 31 December 2019.

We defined treatable and preventable deaths as per avoidable mortality outcomes outlined in the guidance of the Office for National Statistics (ONS),^8^ noting that some causes of death are both treatable and preventable. As per the ONS guidance, avoidable mortality analyses excluded any deaths at the age of 75+ years, thus the following cases were excluded from the avoidable mortality analyses: (1) anyone who was 75+ years at the start of the study (i.e., census day 27 March 2011); (2) anyone who died at the age of 75+ years during the study duration and (3) anyone who turned 75+ years at any point during the study period.

### Data processes

#### All follow-up/censoring

Adults aged 25+ years were followed up from the census date (27 March 2011), and all models were censored on death or the study end date (31 December 2019), whichever came first, unless stated otherwise. Cases of individuals who died either before or on the day of census (i.e., any deaths prior to 28 March 2011) were excluded from the analyses, as participants were expected to spend at least one day in the study to enable us to run survival analysis. Furthermore, in the comparison group, we also excluded individuals who subsequently died during the study period and had their cause of death recorded as a form of an intellectual disability/autism (ICD-10 codes: F70, F71, F72, F73, F78, F79, F84, Q90.0, Q90.1, Q90.2, Q90.9).

#### Missing data

Data linkage was conducted by the National Records of Scotland (NRS). All data provided to us for this study included complete cases only, that is, no observations were included who had missing or imputed cells for any variable. We included all cases provided from NRS in the analysis apart from the exclusions mentioned above. Any errors in cause of death records such as omission, use of abbreviations or ambiguous deaths were listed as an unknown cause.

### Analyses

Information on age, sex and SIMD was recorded on the census day, that is, 27 March 2011. Explorative statistical analyses including t-tests and χ^2^ tests were used to investigate characteristics of adults with intellectual disabilities compared with peers in the general population. Differences in mean age at death were explored using t-tests.

Crude mortality rates (CMRs) per 100 000 were calculated using the censor date/date of death. For indirect standardisation, observed deaths were assumed to be independent and vary with the Poisson distribution. The mortality rates were indirectly standardised for both men and women, using the expected age-specific mortality rates per 1 year age group, using Stata’s ‘strate’ command, to calculate age-SMRs for adults with versus without intellectual disabilities. The 95% CIs were calculated based on the quadratic approximation of the log likelihood. Expected rates were calculated using fixed age-specific and sex-specific rates from the large control population. The SMRs were subsequently calculated stratified by age category (25–34, 35–44, 45–54, 55–64, 65–74 and 75+ years), sex and SIMD.

For all-cause mortality, Kaplan-Meier survival curves were plotted for the study period for both groups, and the proportional hazards assumption was tested.

For the underlying causes of death and all contributing factors in death, the total numbers of deaths in each ICD-10 chapter was collated. We then collated the number of deaths for the top 20 most commonly recorded underlying causes and all contributing factors in death. For cause-specific SMRs, indirect sex-standardisation was also performed (using 10-year age bands). The rates and age-standardised SMRs for avoidable, treatable and preventable mortality were calculated using robust errors.

Cox proportional hazard models were fitted to the data to calculate risks of mortality (all-cause, avoidable, treatable, preventable) unadjusted and adjusted for age, sex and SIMD. For categories which had fewer than 10 deaths, no calculation was attempted due to lack of reliability. Furthermore, in keeping with the ONS mortality methodology, all mortality rates between 10 and 20 deaths were labelled as unreliable.^8^ Two researchers (DN and ER) carried out the main analyses. All analyses were conducted in Stata V.17.

## Results

The study cohort included 14 477 adults with intellectual disabilities aged 25+ and 506 207 adults without intellectual disabilities nor autism aged 25+, following exclusion of 71 individuals with a record of death before or on the date of the census and exclusion of 51 cases without intellectual disabilities or autism who died during the study but had their cause of death recorded as a form of intellectual disability/autism.

### Demographic information

Table 1 presents demographic information on the population of adults with and without intellectual disabilities, at the time of Scotland’s Census, 2011. Compared with their peers, adults with intellectual disabilities had a higher proportion of men (n=7927, 54.8% vs n=238 036, 47.0%; p<0.001), were more likely to be living in more deprived neighbourhoods (p<0.001) and were overall younger (p<0.001).

**Table 1:** Demographic information in 2011* for adults with and without intellectual disabilities

Demographic information*	Intellectual disabilities	Controls	P value†
Total, n (person-years)	14 477 (113 043.63)	506 207 (4 159 808.6)	
Male sex, n (%)	7927 (54.8)	238 036 (47.0)	<0.001
Co-occurring autism			
Total, n (%)	1631 (11.3)	–	
Male sex, n (%)	1017 (7.0)	–	
Age at the time of census, n (%)			
25–34	2974 (20.5)	83 868 (16.6)	<0.001
35–44	3276 (22.6)	97 881 (19.3)	
45–54	3663 (25.3)	108 048 (21.3)	
55–64	2493 (17.2)	92 693 (18.3)	
65–74	1330 (9.2)	67 519 (13.3)	
75+	741 (5.1)	56 198 (11.1)	
SIMD quintile at the time of census, n (%)			
1 (most deprived)	4226 (29.2)	91 378 (18.1)	<0.001
2	3781 (26.1)	98 114 (19.4)	
3	2950 (20.4)	104 062 (20.6)	
4	2209 (15.3)	108 765 (21.5)	
5 (least deprived)	1311 (9.1)	103 888 (20.5)	
Deaths, crude rate per 100 000 (CI)‡	3033.342 (2933.494 to 3136.588)	1674.140 (1661.752 to 1686.62)	

* Data taken from time of Ccensus.

† χ2 test for intellectual disabilities compared with control group (for age and SIMD, χ2 test was performed across all categories, overall p value).

‡ 58 individuals aged 25+ had a record of death which occurred before the date of the census and 13 individuals died on the day of the census; both groups were removed. 51 cases without intellectual disabilities or autism died during the study but had their cause of death recorded as a form of intellectual disability/autism and were subsequently removed from the study.

SIMDScottish Index of Multiple Deprivation

### All-cause mortality

#### Crude mortality

The study period (27 March 2011 to 31st December 2019) resulted in the equivalent of 4 272 853 person years of follow-up. This included 113 044 person years contributed by the intellectual disabilities population and 4 159 809 person years for the population without intellectual disabilities. The median age at death for adults with intellectual disabilities was 65 years (SD=14.1, IQR=56.0–76.0) compared with 80 years (SD=12.9, IQR=71.0–87.0) for adults without intellectual disabilities.

Of the 14 477 adults with intellectual disabilities 3429 (23.7%) died during the 8.5 years of follow-up. In the population without intellectual disabilities, 69 641/506 207 (13.8%) adults died during the same follow-up period. Crude mortality over the study period was 3033.3 (95% CI 2933.5 to 3136.6) per 100 000 person years of follow-up (online supplemental table 1) among adults with intellectual disabilities and 1674.1 (95% CI 1661.8 to 1686.6) for adults without intellectual disabilities (online supplemental table 2). The proportional hazards assumption was visually assessed and met. Kaplan-Meier survival curves for the overall time period were run (online supplemental figure 1).

#### Standardised mortality ratios

For all-cause mortality, compared with adults without intellectual disabilities, the age-standardised SMR in the population with intellectual disabilities was 3.1 (95% CI 3.0 to 3.2). The sex-standardised SMR was 1.8 (95% CI 1.7 to 1.9) and SIMD-standardised SMR was 1.7 (95% CI 1.6 to 1.7) (table 2).

**Table 2:** Standardised mortality ratios (SMRs) for adults with intellectual disabilities compared with controls for age, sex and deprivation (SIMD)

Demographic variables	All deaths, SMR(95% CI)	Avoidable deaths, SMR (95% CI)	Treatable deaths, SMR (95% CI)	Preventable deaths, SMR (95% CI)
Age				
Overall age-SMR	3.130 (3.027 to 3.236)	3.227 (3.041 to 3.425)	4.490 (4.174 to 4.829)	2.511 (2.329 to 2.707)
25–34	6.400 (5.489 to 7.462)	4.196 (3.351 to 5.254)	8.685 (6.463 to 11.671)	2.781 (2.069 to 3.737)
35–44	5.139 (4.589 to 5.755)	3.818 (3.267 to 4.463)	5.676 (4.675 to 6.891)	2.540 (2.051 to 3.145)
45–54	5.299 (4.931 to 5.695)	3.414 (3.070 to 3.795)	4.480 (3.926 to 5.111)	2.715 (2.375 to 3.103)
55–64	4.143 (3.882 to 4.422)	3.015 (2.753 to 3.301)	4.214 (3.780 to 4.698)	2.422 (2.164 to 2.710)
65–74	2.522 (2.344 to 2.713)	1.980 (1.474 to 2.661)	2.652 (1.854 to 3.793)	1.946 (1.397 to 2.711)
75+	1.632 (1.506 to 1.767)	–	–	–
Sex				
Overall sex-SMR	1.808 (1.748 to 1.869)	2.734 (2.576 to 2.901)	3.796 (3.529 to 4.083)	2.095 (1.943 to 2.258)
Male	1.682 (1.605 to 1.762)	2.362 (2.183 to 2.555)	3.269 (2.962 to 3.608)	1.822 (1.655 to 2.005)
Female	1.967 (1.875 to 2.065)	3.453 (3.153 to 3.780)	4.714 (4.229 to 5.253)	2.745 (2.433 to 3.097)
SIMD				
Overall SIMD-SMR	1.688 (1.632 to 1.745)	2.446 (2.305 to 2.596)	3.451 (3.209 to 3.713)	1.878 (1.743 to 2.025)
1 (most deprived)	1.380 (1.296 to 1.470)	1.850 (1.664 to 2.057)	2.565 (2.242 to 2.933)	1.462 (1.283 to 1.666)
2	1.587 (1.487 to 1.693)	2.488 (2.223 to 2.784)	3.573 (3.114 to 4.100)	1.889 (1.638 to 2.179)
3	1.907 (1.773 to 2.052)	3.075 (2.703 to 3.500)	4.172 (3.562 to 4.886)	2.354 (1.996 to 2.777)
4	2.031 (1.863 to 2.215)	2.941 (2.471 to 3.499)	4.298 (3.511 to 5.261)	2.316 (1.855 to 2.892)
5 (least deprived)	2.459 (2.206 to 2.741)	4.356 (3.517 to 5.395)	5.501 (4.253 to 7.116)	3.271 (2.458 to 4.354)

SIMDScottish Index of Multiple Deprivation

The age-stratified SMR was highest in the youngest age group (25–34 years old) at 6.4 (95% CI 5.5 to 7.5) and gradually decreased with age, with the lowest SMR recorded for the oldest age group of 75+ year old at 1.6 (95% CI 1.5 to 1.8). The sex-stratified SMR was higher for women (SMR 2.0, 95% CI 1.9 to 2.1) than men (SMR 1.7, 95% CI 1.6 to 1.8). SMR was also highest in the most affluent areas (SMR=2.5, 95% CI 2.2 to 2.7) and gradually decreased with rising deprivation level, with SMR in the most deprived areas recorded at 1.4 (95% CI 1.3 to 1.5).

### Cause-specific mortality

Table 3 reports the CMRs for underlying causes of death and all contributing factors in death by ICD-10 chapters.

**Table 3:** Crude mortality rates per 100 000 person-years and standardised mortality ratios for underlying causes of death and all contributing factors in death by ICD-10 chapters for adults aged 25+ with and without intellectual disabilities

ICD-10 chapter	Intellectual disabilities		Controls		SMR (95% CI)		
	n (%)	CMR (95% CI)	n (%)	CMR (95% CI)	All	Men	Women
*Underlying cause of death*							
Ch. 1. Certain infectious and parasite diseases	53 (1.5)	46.885 (35.819 to 61.369)	838 (1.2)	20.145 (18.826 to 21.556)	4.144 (3.166 to 5.425)	4.434 (3.100 to 6.341)	3.728 (2.477 to 5.610)
Ch. 2. Neoplasms	489 (14.3)	432.576 (395.886 to 472.667)	20 667 (29.7)	496.826 (490.098 to 503.646)	1.366 (1.250 to 1.492)	1.234 (1.090 to 1.396)	1.510 (1.330 to 1.714)
Ch. 3. Diseases of the blood, blood-forming organs and immune mechanism	<10	–	135 (0.2)	3.245 (2.742 to 3.842)	–	–	–
Ch. 4. Endocrine, nutritional and metabolic diseases	90 (2.6)	79.615 (64.755 to 97.886)	1389 (2.0)	33.391 (31.680 to 35.194)	3.149 (1.501 to 6.605)	3.573 (2.715 to 4.701)	3.919 (2.863 to 5.364)
Ch. 5. Mental and behavioural disorders	243 (7.1)	214.961 (189.564 to 243.761)	5084 (7.3)	122.217 (118.903 to 125.623)	3.919 (3.456 to 4.444)	3.961 (3.283 to 4.778)	3.833 (3.236 to 4.540)
Ch. 6. Diseases of the nervous system	442 (12.9)	390.999 (356.196 to 429.204)	3981 (5.7)	95.702 (92.774 to 98.721)	7.771 (7.080 to 8.531)	7.580 (6.631 to 8.664)	7.842 (6.885 to 8.931)
Ch. 7. Diseases of the eye and adnexa	<5	–	<5	–	–	–	–
Ch. 8. Diseases of the ear and mastoid process	<5	–	<5	–	–	–	–
Ch. 9. Diseases of the circulatory system	692 (20.2)	612.1530 (568.201 to 659.505)	19 093 (27.4)	458.987 (452.523 to 465.544)	2.455 (2.278 to 2.645)	2.220 (2.006 to 2.456)	2.634 (2.360 to 2.941)
Ch. 10. Diseases of the respiratory system	484 (14.1)	428.153 (391.659 to 468.048)	8716 (12.5)	209.529 (205.176 to 213.974)	3.930 (3.595 to 4.296)	4.095 (3.628 to 4.622)	3.696 (3.240 to 4.215)
Ch. 11. Diseases of the digestive system	166 (4.8)	146.846 (126.124 to 170.973)	3616 (5.2)	86.927 (84.139 to 89.807)	2.548 (2.188 to 2.967)	2.328 (1.889 to 2.869)	2.732 (2.189 to 3.411)
Ch. 12. Diseases of the skin and subcutaneous tissue	12 (0.3)^u^	10.615 (6.029 to 18.692)^u^	226 (0.3)	5.433 (4.769 to 6.190)	3.483 (1.978 to 6.133)^u^	–	–
Ch. 13. Diseases of the musculoskeletal system and connective tissue	22 (0.6)	19.461 (12.814 to 29.557)	449 (0.6)	10.794 (9.840 to 11.840)	3.152 (2.075 to 4.787)	4.086 (2.263 to 7.378)^u^	2.631 (1.457 to 4.751)^u^
Ch. 14. Diseases of the genitourinary system	98 (2.9)	86.692 (71.121 to 105.673)	1364 (2.0)	32.790 (31.095 to 34.577)	5.602 (4.595 to 6.828)	6.460 (4.896 to 8.523)	4.919 (3.707 to 6.527)
Ch. 15. Pregnancy, childbirth and puerperium	<5	–	<5	–	–	–	–
Ch. 16. Certain conditions originating in the perinatal period	<5	–	<5	–	–	–	
Ch. 17. Congenital malformations, deformations and chromosomal abnormalities	426 (12.4)	376.846 (342.707 to 414.385)	65 (0.1)	1.563 (1.225 to 1.993)	259.5 (236.0 to 285.4)	224.4 (196.9 to 255.8)	306.5 (267.0 to 351.8)
Ch. 18. Symptoms, signs and abnormal clinical and laboratory findings	56 (1.6)	49.538 (38.124 to 64.371)	936 (1.3)	22.501 (21.105 to 23.990)	3.110 (2.393 to 4.041)	2.240 (1.488 to 3.370)	4.002 (2.845 to 5.629)
Ch. 19. Injury, poisoning and certain other consequences of external causes	<5	–	<5	–	–	–	–
Ch. 20. External causes of morbidity and mortality	115 (3.4)	101.731 (84.738 to 122.131)	2571 (3.7)	61.806 (59.462 to 64.242)	2.118 (1.764 to 2.543)	1.719 (1.353 to 2.185)	2.636 (1.986 to 3.498)
Unknown cause of death or error in underlying cause of death code	<5	–	<5	–	–	–	–
Total number of deaths	3429 (100)	3033.342 (2933.494 to 3136.588)	69 641 (100)	1674.140 (1661.752 to 1686.62)	3.130 (3.027 to 3.236)	2.882 (2.751 to 3.019)	3.329 (3.172 to 3.494)
*All contributing factors in death*							
Ch. 1. Certain infectious and parasite diseases	241 (7.0)	213.192 (187.906 to 241.881)	3774 (5.4)	90.725 (87.877 to 93.667)	3.929 (3.463 to 4.457)	3.848 (3.242 to 4.567)	3.915 (3.248 to 4.719)
Ch. 2. Neoplasms	572 (16.7)	505.999 (466.186 to 549.213)	23 717 (34.1)	570.146 (562.936 to 577.449)	1.422 (1.310 to 1.544)	1.298 (1.159 to 1.454)	1.554 (1.380 to 1.750)
Ch. 3. Diseases of the blood, blood-forming organs and immune mechanism	33 (1.0)	29.192 (20.754 to 41.062)	1001 (1.4)	24.064 (22.618 to 25.601)	2.151 (1.529 to 3.025)	1.566 (0.909 to 2.696)^u^	2.783 (1.796 to 4.314)^u^
Ch. 4. Endocrine, nutritional and metabolic diseases	467 (13.6)	413.115 (377.296 to 452.335)	8672 (12.5)	208.471 (204.129 to 212.905)	3.327 (3.039 to 3.643)	2.706 (2.381 to 3.076)	4.071 (3.581 to 4.629)
Ch. 5. Mental and behavioural disorders	944 (27.5)	835.076 (783.469 to 890.082)	10 899 (15.7)	262.007 (257.134 to 266.973)	6.330 (5.939 to 6.747)	6.151 (5.625 to 6.726)	6.281 (5.734 to 6.880)
Ch. 6. Diseases of the nervous system	943 (27.5)	834.191 (782.612 to 889.170)	7266 (10.4)	174.672 (170.701 to 178.734)	8.890 (8.341 to 9.476)	8.473 (7.747 to 9.267)	9.112 (8.319 to 9.980)
Ch. 7. Diseases of the eye and adnexa	<5	–	41 (0.06)	0.986 (0.726 to 1.339)	–	–	–
Ch. 8. Diseases of the ear and mastoid process	<5	–	11 (0.02)	0.264 (0.146 to 0.477)	–	–	–
Ch. 9. Diseases of the circulatory system	1271 (37.1)	1124.345 (1064.201 to 1187.888)	35 223 (50.6)	846.746 (837.948 to 855.635)	2.459 (2.328 to 2.598)	2.231 (2.069 to 2.406)	2.644 (2.440 to 2.865)
Ch. 10. Diseases of the respiratory system	1616 (47.1)	1429.537 (1361.51 to 1500.962)	24 834 (35.7)	596.999 (589.620 to 604.47)	4.506 (4.291 to 4.731)	4.400 (4.118 to 4.702)	4.506 (4.193 to 4.842)
Ch. 11. Diseases of the digestive system	288 (8.4)	254.769 (226.981 to 285.959)	6824 (9.8)	164.046 (160.200 to 167.985)	2.374 (2.115 to 2.664)	2.248 (1.926 to 2.624)	2.440 (2.051 to 2.904)
Ch. 12. Diseases of the skin and subcutaneous tissue	46 (1.3)	40.692 (30.480 to 54.327)	727 (1.0)	17.477 (16.251 to 18.795)	4.173 (3.126 to 5.572)	3.857 (2.515 to 5.916)	4.443 (3.002 to 6.575)
Ch. 13. Diseases of the musculoskeletal system and connective tissue	62 (1.8)	54.846 (42.761 to 70.347)	1791 (2.6)	43.055 (41.106 to 45.096)	2.324 (1.812 to 2.981)	2.672 (1.845 to 3.870)	2.143 (1.531 to 3.000)
Ch. 14. Diseases of the genitourinary system	379 (11.1)	335.269 (303.159 to 370.780)	8911 (12.8)	214.217 (209.815 to 218.711)	3.150 (2.848 to 3.484)	3.261 (2.840 to 3.744)	2.984 (2.576 to 3.456)
Ch. 15. Pregnancy, childbirth and puerperium	<5	–	<5	–	–	–	–
Ch. 16. Certain conditions originating in the perinatal period	<5	–	<5	–	–	–	–
Ch. 17. Congenital malformations, deformations and chromosomal abnormalities	742 (21.6)	656.384 (610.814 to 705.353)	127 (0.2)	3.053 (2.566 to 3.633)	238.3 (221.8 to 256.1)	207.5 (188.2 to 228.9)	275.2 (247.5 to 306.0)
Ch. 18. Symptoms, signs and abnormal clinical and laboratory findings	521 (15.2)	460.884 (422.961 to 502.208)	9745 (14.0)	234.266 (229.660 to 238.963)	3.808 (3.494 to 4.149)	3.784 (3.342 to 4.284)	3.763 (3.341 to 4.237)
Ch. 19. Injury, poisoning and certain other consequences of external causes	194 (5.7)	171.615 (149.088 to 197.546)	4068 (5.8)	97.793 (94.834 to 100.845)	2.506 (2.177 to 2.885)	2.309 (1.928 to 2.766)	2.585 (2.064 to 3.237)
Ch. 20. External causes of morbidity and mortality	234 (6.8)	207.000 (182.106 to 235.296)	4927 (7.1)	118.443 (115.182 to 121.797)	2.565 (2.257 to 2.916)	2.313 (1.957 to 2.733)	2.749 (2.251 to 3.358)
Unknown cause of death or error in underlying cause of death code	<5	–	<5	–	–	–	–
Total number of deaths	3429 (100)	3033.342 (2933.494 to 3136.588)	69 641 (100)	1674.140 (1661.752 to 1686.62)	3.130 (3.027 to 3.236)	2.882 (2.751 to 3.019)	3.329 (3.172 to 3.494)

CMR rates based on 10–20 deaths labelled “u” for unreliable.

* n<5 repressed due to statistical disclosure.

CMR, crude mortality rate – reported for ≥10 deaths; ICD-10, International Classification of Diseases, Tenth RevisionSMRstandardised mortality ratio

#### Underlying cause of death

The most common underlying causes of death in the adults with intellectual disabilities were: diseases of the circulatory system (n=692; CMR=612.2, 95% CI 568.2 to 659.5); neoplasms (n=489; CMR=432.6, 95% CI 395.9 to 472.7); diseases of the respiratory system (n=484; CMR=428.2, 95% CI 391.7 to 468.0) and diseases of the nervous system (n=442; CMR=391.0, 95% CI 356.2 to 429.2). In the control group without intellectual disabilities, the most common underlying causes of death were: neoplasms (n=20 667; CMR=496.8, 95% CI 490.1 to 503.6); diseases of the circulatory system (n=19 093; CMR=459.0, 95% CI 452.5 to 465.5), diseases of the respiratory system (n=8716; CMR=209.5, 95% CI 205.2 to 214.0) and mental and behavioural disorders (n=5084; CMR=122.2, 95% CI 118.9 to 125.6).

#### All contributing factors in death

In the group with intellectual disabilities, the most common all contributing factors in death were: diseases of the respiratory system (n=1616; CMR=1429.5, 95% CI 1361.5 to 1501.0); diseases of the circulatory system (n=1271; CMR=1124.3, 95% CI 1064.2 to 1187.9); mental and behavioural disorders (n=944; CMR=835.1, 95% CI 783.5 to 890.1) and diseases of the nervous system (n=943; CMR=834.2, 95% CI 782.6 to 889.2). In the control group without intellectual disabilities, the most common were diseases of the circulatory system (n=35 223; CMR=846.7, 95% CI 837.9 to 855.6), the respiratory system (n=24 834; CMR=597.0, 95% CI 589.6 to 604.5) and neoplasms (n=23 717; CMR=570.1, 95% CI 562.9 to 577.4).

#### Most common causes of death

Table 4 reports the most common individual underlying causes and all contributing factors in death. Based on pre-specified groupings of specific ICD-10 codes, among adults with intellectual disabilities, the most commonly recorded underlying causes of death were Down syndrome, dementia and acute myocardial infarction. For all contributing factors in death, pneumonia due to organism unspecified, Down syndrome and pneumonitis due to solids and liquids were the most commonly recorded. In the population of adults without intellectual disabilities, the most commonly recorded underlying causes of death were malignant neoplasm of bronchus and lung, acute myocardial infarction and dementia. For all contributing factors in death, chronic ischaemic heart disease, pneumonia due to organism unspecified and other chronic obstructive pulmonary disease were most commonly recorded.

**Table 4:** Most common causes of death in adults with and without intellectual disabilities

Intellectual disabilities		Controls	
Cause (n)	ICD codes	Cause (n)	ICD codes
*Underlying causes of deaths*			
1. Down Syndrome (n=341)	Q90.0, Q90.1, Q90.2, **Q90.9**	Malignant neoplasm of bronchus and lung (n=5163)	**C34.0, C34.1, C34.2, C34.3, C34.8, C34.9**
2. Dementia (n=227)	F00.0, F00.1, F00.2, F00.9, **F03, G30.0, G30.1**, G30.8, **G30.9**	Acute myocardial infarction (n=4907)	I21.0, I21.1, I21.2, I21.3, **I21.4, I21.9**
3. Acute myocardial infarction (n=158)	I21.0, I21.1, I21.2, I21.3, I21.4, **I21.9**	Dementia (n=4857)	F00.0, F00.1, F00.2, F00.9, **F03, G30.0**, **G30.1**, G30.8, **G30.9**
4. Pneumonia, organism unspecified (n=150)	J18.0, J18.1, J18.2, J18.8, **J18.9**	Other chronic obstructive pulmonary disease (n=3720)	**J44.0, J44.1, J44.8, J44.9**
5. Epilepsy (n=137)	G40.0, G40.1, G40.2, G40.3, G40.4, G40.5, **G40.6**, G40.7, G40.8, **G40.9**, G41.0, G41.1, G41.2, G41.8, **G41.9**	Chronic ischaemic heart disease (n=3672)	**I25.0, I25.1**, I25.2, **I25.3, I25.4, I25.5**, I25.6, I25.7, **I25.8, I25.9**
6. Pneumonitis due to solids and liquids (n=128)	**J69.0**, J69.1, J69.8	Intracerebral haemorrhage, cerebral infarction and stroke, not specified as haemorrhage or infarction (n=2814)	**I61.0, I61.2, I61.3, I61.4, I61.5, I61.6, I61.8, I61.9, I63.0**, I63.1, **I63.2, I63.3, I63.4, I63.5**, I63.6, **I63.8, I63.9, I64**
7. Cerebral palsy (n=123)	**G80.0, G80.1**, G80.2, G80.3, G80.4, **G80.8**, **G80.9**	Pneumonia, organism unspecified (n=2194)	**J18.0, J18.1, J18.2**, J18.8, J18.9
8. Intracerebral haemorrhage, cerebral infarction and stroke, not specified as haemorrhage or infarction (n=111)	**I61.0**, I61.2, I61.3, **I61.4**, I61.5, **I61.6**, I61.8, **I61.9**, I63.0, I63.1, I63.2, **I63.3**, I63.4, **I63.5, I63.6**, I63.8, **I63.9, I64**	Vascular dementia (n=2136)	F01.0, **F01.1, F01.2, F01.3**, F01.8, **F01.9**
9. Chronic ischaemic heart disease (n=110)	**I25.0, I25.1**, I25.2, I25.3, I25.4, I25.5, I25.6, I25.7, **I25.8, I25.9**	Malignant neoplasm of breast unspecified (n=1303)	C50.0, C50.1, C50.2, C50.3, C50.5, C50.6, C50.8, **C50.9**
10. Other chronic obstructive pulmonary disease (n=78)	**J44.0, J44.1, J44.8, J44.9**	Malignant neoplasm of prostate (n=1237)	**C61**
11. Malignant neoplasm of bronchus and lung (n=67)	**C34.0, C34.1**, C34.2, **C34.3**, C34.8, **C34.9**	Accidents: other external causes of accidental injury: Falls (n=1005)	**W19**
12. Vascular dementia (n=61)	F01.0, **F01.1**, F01.2, F01.3, F01.8, **F01.9**	Malignant neoplasm of oesophagus (n=999)	**C15.9**
13. Urinary tract infection (n=48)	**N39.0**	Sequelae of other and unspecified cerebrovascular diseases (n=996)	**I69.8**
14. Other specified respiratory disorders (n=41)	**J98.8**	Malignant neoplasm: pancreas (n=886)	**C25.9**
15. Other ill identified cause of mortality (n=38)	**R99**	Malignant neoplasm: colon (n=728)	**C18.9**
16. Sequelae of other and unspecified cerebrovascular diseases (n=37)	**I69.8**	Urinary tract infection (n=638)	**N39.0**
17. Malignant neoplasm of oesophagus (n=33)	**C15.9**	Malignant neoplasm: bladder (n=628)	**C67.9**
18. Malignant neoplasm of breast (n=30): C50.0, C50.1, C50.2, C50.3, C50.5, C50.6, C50.8, **C50.9** Malignant neoplasm, site unspecified (n=30): **C80**		Malignant neoplasm, site unspecified (n=594)	**C80**
19. Developmental disorder of scholastic skills, unspecified (n=28)	**F81.9**	Other interstitial pulmonary diseases with fibrosis (n=576)	**J84.1**
20. Sepsis, unspecified (n=26)	**A41.9**	Parkinson disease (n=554)	**G20**
*All contributing factors in deaths*			
1. Pneumonia, organism unspecified (n=731)	**J18.0, J18.1, J18.2**, J18.8, **J18.9**	Chronic ischaemic heart disease (n=12 607)	**I25.0, I25.1**, I25.2, **I25.3, I25.4, I25.5**, I25.6, I25.7, **I25.8, I25.9**
2. Down syndrome (n=593)	Q90.0, Q90.1, Q90.2, **Q90.9**	Pneumonia, organism unspecified (n=9833)	**J18.0, J18.1, J18.2, J18.8, J18.9**
3. Pneumonitis due to solids and liquids (n=487)	**J69.0**, J69.1, J69.8	Other chronic obstructive pulmonary disease (n=9012)	**J44.0, J44.1, J44.8, J44.9**
4. Epilepsy (n=412)	G40.0, G40.1, **G40.2, G40.3, G40.4**, G40.5, **G40.6**, G40.7, G40.8, **G40.9**, G41.0, G41.1, G41.2, G41.8, **G41.9**	Dementia (n=8474)	F00.0, F00.1, F00.2, F00.9, **F03, G30.0, G30.1, G30.8, G30.9**
5. Developmental disorder of scholastic skills, unspecified (n=380)	**F81.9**	Acute myocardial infarction (n=5968)	**I21.0**, I21.1, I21.2, I21.3, **I21.4, I21.9**
6. Dementia (n=357)	F00.0, F00.1, F00.2, F00.9, **F03, G30.0, G30.1**, G30.8, **G30.9**	Essential primary hypertension (n=5879)	**I10.0**
7. Chronic ischaemic heart disease (n=295)	**I25.0, I25.1**, I25.2, I25.3, I25.4, I25.5, I25.6, I25.7, **I25.8**, I25.9	Malignant neoplasm of bronchus and lung (n=5727)	**C34.0, C34.1, C34.2, C34.3, C34.8, C34.9**
8. Cerebral palsy (n=215)	**G80.0, G80.1**, G80.3, G80.4, **G80.8, G80.9**	Other general symptoms and signs (n=5058)	**R68.8**
9. Acute myocardial infarction (n=202)	I21.0, I21.1, I21.2, I21.3, I21.4, **I21.9**	Intracerebral haemorrhage, cerebral infarction and stroke, not specified as haemorrhage or infarction (n=4733)	**I61.0, I61.1, I61.2, I61.3, I61.4, I61.5, I61.6, I61.8, I61.9, I63.0**, I63.1, **I63.2, I63.3, I63.4, I63.5, I63.6, I63.8, I63.9, I64**
10. Diabetes without complications (n=196)	**E11.9**	Diabetes without complications (n=4495)	**E11.9**
11. Other general symptoms and signs (n=185)	**R68.8**	Vascular dementia (n=3948)	F01.0, **F01.1, F01.2, F01.3**, F01.8, **F01.9**
12. Intracerebral haemorrhage, cerebral infarction and stroke, not specified as haemorrhage or infarction (n=178)	**I61.0**, I61.1, I61.2, I61.3, **I61.4, I61.5, I61.6**, I61.8, **I61.9**, I63.0, I63.1, **I63.2, I63.3**, I63.4, **I63.5, I63.6, I63.9, I64**	Pneumonitis due to solids and liquids (n=2945)	**J69.0**, J69.1, **J69.8**
13. Sepsis, unspecified (n=173)	**A41.9**	Atrial fibrillation and flutter (n=2734)	**I48**
14. Other chronic obstructive pulmonary disease (n=165)	J44.0, **J44.1, J44.8, J44.9**	Sepsis, unspecified (n=2522)	**A41.9**
15. Essential primary hypertension (n=130)	**I10**	Age-related physical debility (n=2513)	**R54**
16. Unspecified acute lower respiratory infection (n=127)	**J22**	Congestive heart failure (n=2439)	**I50.0**
17. Urinary tract infection site not specified (n=113)	**N39.0**	Sequelae of other and unspecified cerebrovascular diseases (n=2346)	**I69.8**
18. Acute renal failure unspecified (n=86)	**N17.9**	Unspecified acute lower respiratory infection (n=2229)	**J22**
19. Congestive heart failure (n=82)	**I50.0**	Atrial fibrillation and atrial flutter, unspecified (n=2213)	**I48.9**
20. Other respiratory disorders (n=81)	**J98.8**	Acute renal failure, unspecified (n=2190)	**N17.9**

While all codes included in the ICD-10 groupings are listed above, the highlights in **bold** refer to those codes, which were present in the data.

ICDInternational Classification of Diseases

### Avoidable mortality

#### Incidence

Of the 3429 deaths recorded for the population of adults with intellectual disabilities, 1087 (31.7%) were considered avoidable. Of all deaths, 722 (21.1%) were treatable and 681 (19.9%) were preventable. In the population of adults without intellectual disabilities, 12 673 (18.2%) of the 69 641 deaths were considered avoidable; 6110 (8.8%) treatable and 10 207 (14.7%) preventable (table 5).

**Table 5:** All-cause, avoidable, treatable and preventable underlying cause of deaths for adults with and without intellectual disabilities

Variable	Deaths in a group with intellectual disabilities (n)				Deaths in controls (n)				HR (95% CI)
	All	Avoidable*	Treatable*	Preventable*	All	Avoidable*	Treatable*	Preventable*	†a (All)†b (Avoidable)†c (Treatable)†d (Preventable)
Total	3429	1087	722	681	69 641	12 673	6110	10 207	**†a** 3.304 (3.192 to 3.420)**2.978 (2.877 to 3.084)**†b** 3.350 (3.148 to 3.564)**2.701 (2.538 to 2.876)**†c** 4.672 (4.325 to 5.048)**3.903 (3.609 to 4.220)**†d** 2.611 (2.416 to 2.822)**2.042 (1.889 to 2.208)
Age									
25–34	163	76	44	44	734	522	146	456	**†a** 3.324 (3.211 to 3.440)**†b** 3.351 (3.149 to 3.565)**†c** 4.674 (4.326 to 5.050)**†d** 2.611 (2.416 to 2.822)
35–44	300	158	102	84	1793	1271	552	1016	
45–54	740	342	221	215	4410	3164	1558	2501	
55–64	907	467	325	303	9369	6630	3301	5355	
65–74	717	44	30	35	17 169	1086	553	879	
75+	602	0	0	0	36 166	0	0	0	
Sex									
Male	1784	619	395	417	33 222	7413	3418	6475	**†a** 3.259 (3.148 to 3.373)**†b** 3.274 (3.077 to 3.483)**†c** 4.597 (4.255 to 4.967)**†d** 2.522 (2.333 to 2.726)
Female	1645	468	327	264	36 419	5260	2692	3732	
SIMD									
1 (most deprived)	962	342	213	226	15 469	3738	1679	3125	**†a** 3.019 (2.916 to 3.125)**†b** 2.768 (2.600 to 2.946)**†c** 3.970 (3.671 to 4.292)**†d** 2.119 (1.960 to 2.291)
2	908	304	203	189	15 467	2938	1366	2405	
3	721	230	154	141	14 127	2490	1229	1994	
4	512	127	94	78	13 159	2020	1023	1575	
5 (least deprived)	326	84	58	47	11 419	1487	813	1108	

Reference groups: no intellectual disabilities, male, most deprived, age (continuous).

* Deaths at age 25–74 only as per the ONS definition of avoidable mortality.

† a-d - Cox regression hazard ratioHR for risk of deaths (all, avoidable, treatable and preventable) by intellectual disabilities versus controls (total column=unadjusted and **adjusted for age, sex and SIMD).

SIMD, Scottish Index of Multiple Deprivation

Crude avoidable mortality in adults with intellectual disabilities was 1061.4 (95% CI 1000.1 to 1126.4) per 100 000 person years of follow-up and 375.9 (95% CI 369.4 to 382.5) for adults without intellectual disabilities. Treatable mortality in the intellectual disabilities group was 705.0 (95% CI 655.4 to 758.3) per 100 000 person years of follow-up and 181.2 (95% CI 176.7 to 185.8) for adults without intellectual disabilities. Preventable mortality for the intellectual disabilities cohort was 665.0 (95% CI 616.9 to 716.8) per 100 000 person years of follow-up and 302.8 (95% CI 296.9 to 308.7) for adults without intellectual disabilities. Further details are provided in online supplemental tables 1 and 2.

#### Standardised mortality ratios

Table 2 shows that SMRs for individual age groups were highest in the youngest age groups for all avoidable deaths as well as for treatable and preventable mortality. SMRs for avoidable, treatable and preventable deaths were higher for females (avoidable: 3.5, 95% CI 3.2 to 3.8; treatable: 4.7, 95% CI 4.2 to 5.3; preventable: 2.7, 95% CI 2.4 to 3.1) than males (avoidable: 2.4, 95% CI 2.2 to 2.6; treatable: 3.3, 95% CI 3.0 to 3.6; preventable: 1.8, 95% CI 1.7 to 2.0). There was a gradient of increasing SMRs for avoidable, treatable and preventable mortality as the extent of the neighbourhood deprivation decreased.

Table 5 reports underlying cause of death with the Cox proportional hazards, unadjusted (HR) and adjusted for age, sex and SIMD (aHR). For all deaths: HR=3.3 (95% CI 3.2 to 3.4) and aHR=3.0 (95% CI 2.9 to 3.1). For avoidable deaths: HR=3.4 (95% CI 3.1 to 3.6), treatable deaths: HR=4.7 (95% CI 4.3 to 5.0) and preventable deaths: HR=2.6 (95% CI 2.4 to 2.8), and aHRs were: avoidable deaths: aHR=2.7 (95% CI 2.5 to 2.9), treatable deaths: aHR=3.9 (95% CI 3.6 to 4.2) and preventable deaths: aHR=2.0 (95% CI 1.9 to 2.2) (table 5).

## Discussion

### Summary of principal findings

For all-cause mortality, compared with adults without intellectual disabilities, the age-standardised SMR in the population with intellectual disabilities was 3.1. The SMRs were higher for the youngest age groups, women and in the most affluent areas. This gradient in increase in the SMRs in more affluent neighbourhoods is likely caused by the difference in the general population across extent of neighbourhood deprivation, rather than a difference across neighbourhoods in the population with intellectual disabilities, that is, the general population experience higher rates of deaths in the more deprived areas whereas for adults with intellectual disabilities this trend is not as pronounced. Adults with intellectual disabilities also died younger than adults without intellectual disabilities (median age at death: 65.0 years vs 80.0 years).

Avoidable deaths were substantially more common in people with intellectual disabilities than other people, particularly due to deaths from conditions that could have been treated with good quality care. Our paper is novel in investigating avoidable deaths in detail, including reporting SMRs for avoidable, treatable and preventable deaths by the sociodemographic features of age, sex and extent of neighbourhood deprivation. SMRs for avoidable deaths ranged from 4.4 to 2.0, being higher at younger age groups, in women and in more affluent neighbourhoods.

For those with intellectual disabilities, the most common underlying causes of death were diseases of the circulatory system, neoplasms, diseases of the respiratory system and diseases of the nervous system, with fairly similar number of deaths recorded in each category. This differs from the controls, where neoplasms were markedly more common, followed by diseases of the circulatory system and much less common diseases of the respiratory system and mental and behavioural disorders. Most commonly recorded all contributing factors in death for adults with intellectual disabilities were diseases of the respiratory system, followed by diseases of the circulatory system, and equally mental and behavioural disorders and diseases of the nervous system. In the control group, the most common all contributing factors in death were diseases of the circulatory system by far. Sex-standardised SMRs for underlying causes of deaths ranged from 259.5 (congenital malformations, deformations and chromosomal abnormalities) to 1.4 (neoplasms). For all contributing factors in deaths, the SMRs ranged from 238.3 (congenital malformations, deformations and chromosomal abnormalities) to 1.4 (neoplasms).

### Comparison with existing literature

We are aware of only three studies on avoidable mortality in adults with intellectual disabilities. These covered shorter periods of time and/or included smaller sample sizes, limiting opportunities for comparison.^2 4 6^ We report that 31.7% of deaths of adults with intellectual disabilities were avoidable, compared with 46.3%,^4^ 38.9%^2^ and 31.0%.^6^ Compared with the general population, we found much higher rates of avoidable (SMR=3.2), treatable (SMR=4.5) and preventable (SMR=2.5) deaths. The incidence rate ratio (IRR) for avoidable deaths reported by Trollor *et al*,^6^ while raised, showed a much lesser difference in avoidable mortality than in our study (IRR 1.47; 95% CI 1.54 to 1.99; p<0.001). Hosking *et al*^4^ reported hazard ratios that were more similar to our findings: avoidable (3.44), treatable (5.86) and preventable (1.69) deaths. Cooper *et al*^2^ did not calculate comparisons with the general population. We are not aware of any previous studies which would have reported on avoidable, treatable and preventable mortality in relation to sociodemographic factors such as age, sex or neighbourhood deprivation with which we could compare our findings.

The high rate of SMR (3.1) we report for all-cause mortality is consistent with previous literature, as is the higher SMR in females, and at younger ages. However, we are not aware of previous studies of SMR in adults with intellectual disabilities in relation to the extent of neighbourhood deprivation. One Australian study reported contrary results to ours and other studies, with regards to sex and age findings.^6^

Few studies have reported causes of death by ICD-10 chapter, and reports are contradictory. By ICD-10 chapter, we found the most common underlying causes of death to be diseases of the circulatory system, neoplasms, diseases of the respiratory system and diseases of the nervous system, similarly to the report by Cooper *et al*^2^ while others reported the most common to be circulatory,^4^ vascular,^19^ heart^20^ and jointly circulatory diseases and neoplasms.^6^ With regard to findings from the analysis of pre-specified groupings of specific ICD-10 codes, we have found that Down syndrome was the most commonly recorded underlying cause of death for adults with intellectual disabilities. This suggests that there may still exist prevailing uncertainty in relation to underlying causes of death in people with intellectual disabilities and that there is continued conflation of disability and health among attending medical practitioners responsible for recording causes of death in Scotland.^21^

### Implications for policy and practice

The higher risk of all-cause, avoidable, treatable and preventable mortality, and earlier age at death for adults with intellectual disabilities than their peers without intellectual disabilities demonstrates a clear need for improvements in the early detection, prevention, care and treatment of health problems experienced by people with intellectual disabilities. This is essential at all ages, and for people living in all areas; more so than for the general population, avoidable mortality in people with intellectual disabilities is not related to older age or neighbourhood deprivation. Recording of Down syndrome as a cause of death in adults with intellectual disabilities is still common among attending medical practitioners in Scotland. It is, therefore, crucial that we better understand how individual health conditions impact on the health and mortality of adults with intellectual disabilities. Research efforts should be directed particularly towards the management of epilepsy and pneumonia to reduce premature mortality due to the diseases of the nervous and respiratory systems, which are one of the leading causes of death in this population. Our findings on mortality caused by cardiovascular diseases and neoplasms in adults with intellectual disabilities also suggest that public health interventions aimed at circulatory diseases and cancer screening may need appropriate adaptation and tailoring for this population. Clinical and health training initiatives should be introduced across all age groups and all neighbourhoods, given that our findings suggest that mortality risk is highest in the most affluent areas for adults with intellectual disabilities.

### Strengths and limitations

A major strength of this study is that it includes a whole country population of adults with intellectual disabilities and a representative proportion of people in the general population, with a high response rate of 94% for Scotland’s Census 2011,^16^ thereby reducing the study bias. Whether each individual had intellectual disabilities was enquired about, and intellectual disabilities were specifically distinguished from specific learning disabilities, and from autism. Prior to the census, these questions were field tested to check their utility and acceptability, using cognitive question testing with 70 respondents on the whole questionnaire and 102 respondents specifically on the health questions. Additionally, the prevalence of intellectual disabilities in the census data (0.5%) is the same as that found in the Scottish General Practice registers, and in other large data sources, which have been used to identify adults with intellectual disabilities.^22^

Limitations include the fact that the census data do not specify whether a record of intellectual disabilities was reported by a person with intellectual disabilities or their proxy (e.g., a parent/carer, spouse, etc). Moreover, respondents reported whether or not each person was known to have intellectual disabilities rather than each person having an assessment for intellectual disabilities, so some reporting error is possible. Further, our death data were taken from death certificates and were not verified at postmortem. The death certificates will have been completed by numerous clinicians and there may be some error in reporting, including between underlying causes and all contributing factors in death, but such error in reporting mostly poses limitations only when investigating more granular outcomes. Some data repression was necessary where very small numbers were identified to mitigate the risk of disclosure. In keeping with the ONS methodology for investigating avoidable mortality,^8^ all CMRs per 100 000 people based on fewer than 20 deaths were labelled as unreliable to warn users of their low reliability. It is also important to note that the ONS list of avoidable deaths is based on general population data and is, therefore, possibly an underestimate of avoidable deaths in the population with intellectual disabilities due to differing health and death profiles.

Given the strengths of the study, we believe the results to be generalisable to other high-income countries, as well as filling a significant gap in existing research on avoidable mortality in adults with intellectual disabilities, and contradictory reports on causes of death. However, this needs to be determined by replication of our findings.

## supplementary material

10.1136/bmjopen-2024-089962 online supplemental file 1

## Data Availability

No data are available.
