# Supplementary material for: Rates, causes and predictors of all-cause and avoidable mortality in 514 878 adults with and without intellectual disabilities in Scotland: a record linkage national cohort study
Source: BMJ Open. 2025 Feb 12;15(2):e089962. doi: 10.1136/bmjopen-2024-089962 (PMC11822423; doi:10.1136/bmjopen-2024-089962)

Supplemental Table 1. Crude mortality rates (CMRs) for adults with intellectual disabilities per 100,000 by age, sex, and deprivation (SIMD)

| Variable | All deaths  CMR (95% CI) | Avoidable deaths  CMR (95% CI) | Treatable deaths  CMR (95% CI) | Preventable deaths  CMR (95% CI) |
| --- | --- | --- | --- | --- |
| All deaths | 3033.342 (2933.494-3136.588) | 1061.407 (1000.147-1126.418) | 705.000 (655.407-758.347) | 664.966 (616.852-716.832) |
| Age | | | | |
| 25-34 | 641.267 (550.006-747.670) | 298.996 (238.795-374.373) | 173.103 (128.819-232.609) | 173.103 (128.819-232.609) |
| 35-44 | 1082.162 (966.380-1211.815) | 569.939 (487.652-666.11) | 367.935 (303.033-446.738) | 303.005 (244.668-375.253) |
| 45-54 | 2508.555 (2334.172-2695.966) | 1159.359 (1042.774-1288.979) | 749.177 (656.638-854.756) | 728.837 (637.645-833.071) |
| 55-64 | 4980.093 (4666.312-5314.974) | 2564.171 (2341.845-2807.605) | 1784.488 (1600.654-1989.435) | 1663.691 (1486.526-1861.971) |
| 65-74 | 8168.600 (7592.048-8788.937) | 2822.336 (2100.319-3792.557) | 1924.320 (1345.457-2752.23) | 2245.040 (1611.925-3126.823) |
| 75+ | 17634.187 (16280.32-19100.64) | - | - | - |
| Sex | | | | |
| Male | 2858.277 (2728.673-2994.036) | 1080.500 (998.648-1169.062) | 689.495 (624.745-760.957) | 727.898 (661.282-801.224) |
| Female | 3249.165 (3095.885-3410.034) | 1037.165 (947.329-1135.519) | 724.686 (650.247-807.646) | 585.067 (518.583-660.076) |
| SIMD | | | | |
| 1 (most deprived) | 2889.038 (2712.123-3077.493) | 1128.080 (1014.64-1254.202) | 702.576 (614.285-803.557) | 745.456 (654.336-849.265) |
| 2 | 3075.061 (2881.414-3281.722) | 1136.144 (1015.345-1271.315) | 758.675 (661.17-870.559) | 706.353 (612.500-814.587) |
| 3 | 3148.364 (2926.743-3386.768) | 1107.029 (972.820-1259.754) | 741.228 (632.937-868.048) | 678.657 (575.395-800.451) |
| 4 | 2965.524 (2719.464-3233.848) | 818.743 (688.044-974.271) | 605.999 (495.082-741.766) | 502.850 (402.772-627.796) |
| 5 (least deprived) | 3243.259 (2909.631-3615.142) | 928.258 (749.540-1149.589) | 640.940 (495.506-829.060) | 519.383 (390.236-691.270) |

Abbreviations: CMR=Crude Mortality Rate; CI=confidence interval

Supplemental Table 2. Crude mortality rates (CMRs) for adults without intellectual disabilities per 100,000 by age sex, and deprivation (SIMD)

| Variable | All deaths  CMR (95% CI) | Avoidable deaths  CMR (95% CI) | Treatable deaths  CMR (95% CI) | Preventable deaths  CMR (95% CI) |
| --- | --- | --- | --- | --- |
| All deaths | 1674.140 (1661.752-1686.62) | 375.904 (369.416-382.506) | 181.234 (176.746-185.835) | 302.758 (296.941-308.689) |
| Age | | | | |
| 25-34 | 100.202 (93.209-107.719) | 71.260 (65.402-77.643) | 19.931 (16.947-23.441) | 62.251 (56.791-68.235) |
| 35-44 | 210.565 (201.041-220.540) | 149.263 (141.278-157.698) | 64.825 (59.637-70.465) | 119.316 (112.201-126.883) |
| 45-54 | 473.375 (459.608-487.554) | 339.628 (327.998-351.670) | 167.238 (159.136-175.752) | 268.460 (258.143-279.191) |
| 55-64 | 1201.950 (1177.857-1226.536) | 850.563 (830.334-871.286) | 423.486 (409.283-438.182) | 686.994 (668.838-705.642) |
| 65-74 | 3239.052 (3190.963-3287.866) | 1425.095 (1342.809-1512.424) | 725.670 (667.640-788.744) | 1153.461 (1079.674-1232.291) |
| 75+ | 10808.359 (10697.54-10920.33) | - | - | - |
| Sex | | | | |
| Male | 1699.664 (1681.485-1718.039) | 457.480 (447.184-468.014) | 210.936 (203.982-218.127) | 399.593 (389.978-409.446) |
| Female | 1651.515 (1634.641-1668.564) | 300.410 (292.4-308.639) | 153.746 (148.046-159.665) | 213.142 (206.413-220.092) |
| SIMD | | | | |
| 1 (most deprived) | 2092.940 (2060.217-2126.183) | 609.863 (590.622-629.730) | 273.933 (261.138-287.354) | 509.851 (492.285-528.043) |
| 2 | 1938.103 (1907.799-1968.889) | 456.686 (440.468-473.502) | 212.333 (201.366-223.897) | 373.836 (359.190-389.079) |
| 3 | 1650.828 (1623.829-1678.276) | 359.964 (346.099-374.384) | 177.669 (168.009-187.885) | 288.260 (275.882-301.194) |
| 4 | 1460.076 (1435.342-1485.237) | 278.420 (266.540-290.831) | 141.002 (132.621-149.913) | 217.085 (206.625-228.075) |
| 5 (least deprived) | 1319.1242 (1295.15-1343.542) | 213.08986 (202.5298-224.2005) | 116.50441 (108.7651-124.7945) | 158.778 (149.699-168.408) |

Abbreviations: CMR=Crude Mortality Rate; CI=confidence interval

Supplemental Figure 1. Kaplan-Meier survival analysis for risk of death for intellectual disabilities population and controls from 2011-2019


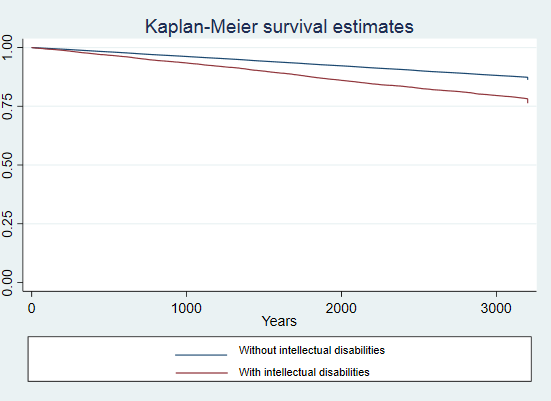

Supplement: online supplemental file 1 [file bmjopen-15-2-s001.docx]
