# Supplementary material for: Rates, causes and predictors of all-cause and avoidable mortality in 514 878 adults with and without intellectual disabilities in Scotland: a record linkage national cohort study
Source: BMJ Open. 2025 Feb 12;15(2):e089962. doi: 10.1136/bmjopen-2024-089962 (PMC11822423; doi:10.1136/bmjopen-2024-089962)
Supplement: Supplementary file 2 [file bmjopen-2024-089962.reviewer_comments.pdf]

PEER REVIEW HISTORY

BMJ Open publishes all reviews undertaken for accepted manuscripts. Reviewers are asked to complete a checklist review form and are provided with free text boxes to elaborate on their assessment. These free text comments are reproduced below.

ARTICLE DETAILS

Title (Provisional)

Rates, causes and predictors of all-cause and avoidable mortality in 514,878 adults with and without intellectual disabilities in Scotland: a record linkage national cohort study

Authors

Rydzewska, Ewelina; Nijhof, Dewy; Hughes-McCormack, Laura; Melville, Craig; Fleming, Michael; Mackay, Daniel; Sosenko, Filip; Ward, Laura McKernan; Dunn, Kirsty; Truesdale, Maria; Cairns, Deborah; Pell, Jill; Wyper, Grant M. A.; Jani, Bhautesh; Barlow, Fiona; Henderson, Angela; Callander, Ruth; Cooper, Sally-Ann

---

VERSION 1 - REVIEW

**Reviewer**	**1**
**Name**	**Srasuebkul, Preeyaporn**
**Affiliation**	**UNSW Australia, Department of Developmental Disability Neuropsychiatry**
**Date**	**23-Aug-2024**
**COI**	**I don't have any competing interests.**

Abstract

1. If possible having the death rate per person-time could be informative too

2. Please spell out SMR first; you did not mention it anywhere, and then use this term in the result.

Methods

3. While researchers gave details about the question for disability, I wonder whether a person can choose not to answer these questions.

4. Researchers should exclude people who died from Down syndrome from the comparison group, if there is any. The ICD-10 codes provided do not include the code for Down syndrome.

My general comment:

5. The authors stated that the prevalence of intellectual disability in Scotland is approximately 0.5%, it seems pretty low to me. Will you be able to offer some explanations?

I want to congratulate the authors on a well-written paper, it is easy to follow, and I can follow the logic with no issues.

**Reviewer**	**2**
**Name**	**Shooshtari , Shahin**
**Affiliation**	**University of Manitoba Department of Community Health Sciences**
**Date**	**23-Oct-2024**
**COI**	**There are no competing interests.**

This is an exceptionally well-written manuscript on a critical topic, supported by a well-designed study. The findings are thoroughly reported and thoughtfully discussed.

To strengthen the manuscript, I suggest including relevant studies conducted in Canada, as they could provide additional context and insights that align with the topic. Incorporating a broader geographical scope would enhance the comprehensiveness of the literature review provided.

VERSION 1 - AUTHOR RESPONSE

Reviewers' Comments to Author:

Reviewer: 1

Abstract
1. If possible, having the death rate per person-time could be informative too

Response: We have now added this information in the abstract (changes highlighted in yellow).

2. Please spell out SMR first; you did not mention it anywhere, and then use this term in the result.

Response: We have made the suggested change.

Methods
3. While researchers gave details about the question for disability, I wonder whether a person can choose not to answer these questions.

Response: We thank the reviewer for this comment. We state in the text (Methods) the following:

‘It was a legal requirement to complete the census, and non-compliance or supplying false information could result in a fine of £1,000. The Census team followed up non-responders and also provided help in responding when it was needed; hence, the high 94% completion rate.’

We of course do not know how truthful the respondents were in their responses. However, Question 20 did not include response options such as ‘Don’t know’ or ‘Prefer not to say’, which would potentially allow respondents not to report having a condition/disability. The response options, which were available to respondents in this question are listed in the manuscript (Methods):

‘Respondents were given a choice of 10 response options, with option number (3) ‘learning disability (e.g., Down’s syndrome)’ being synonymous in the UK with the international term ‘intellectual disabilities’. The remaining response options were as follows: (1) deafness or partial hearing loss, (2) blindness or partial sight loss, (4) learning difficulty (e.g., dyslexia), (5) developmental disorder (e.g., autistic spectrum disorder or Asperger’s syndrome), (6) physical disability, (7) mental health condition, (8) long-term illness, disease or condition, (9) other condition, (10) no condition.’

4. Researchers should exclude people who died from Down syndrome from the comparison group, if there is any. The ICD-10 codes provided do not include the code for Down syndrome.

Response: We thank the reviewer for spotting this omission in the text, which we have now corrected as follows:

‘Furthermore, in the comparison group, we also excluded individuals who subsequently died during the study period and had their cause of death recorded as a form of an intellectual disability/autism (ICD-10 codes: F70, F71, F72, F73, F78, F79, F84, Q90.0, Q90.1, Q90.2, Q90.9).’

My general comment:
5. The authors stated that the prevalence of intellectual disability in Scotland is approximately 0.5%, it seems pretty low to me. Will you be able to offer some explanations?

Response: We thank the reviewer for this comment. As mentioned in our response to comment number 3, Scotland’s Census had a 94% completion rate. Given that respondents were instructed that non-compliance or supplying false information could result in a fine of £1,000, we believe that the collected data are a true representation of the prevalence rate of intellectual disabilities at the time. We of course have no way to verify this further.

We do, however, state in the section ‘Strengths and Limitations’ the following:

‘Additionally, the prevalence of intellectual disabilities in the Census data (0.5%) is the same as that found in Scottish GP registers, and in other large data sources, which have been used to identify adults with intellectual disabilities.[19]’

‘(…) respondents reported whether or not each person was known to have intellectual disabilities rather than each person having an assessment for intellectual disabilities, so some reporting error is possible.’

I want to congratulate the authors on a well-written paper, it is easy to follow, and I can follow the logic with no issues.

Response: We thank the reviewer for these kind words and for their expertise and time spent reviewing the manuscript.

Reviewer: 2

This is an exceptionally well-written manuscript on a critical topic, supported by a well-designed study. The findings are thoroughly reported and thoughtfully discussed.
To strengthen the manuscript, I suggest including relevant studies conducted in Canada, as they could provide additional context and insights that align with the topic. Incorporating a broader geographical scope would enhance the comprehensiveness of the literature review provided.

Response: We thank the reviewer for these kind words and for their expertise and time spent reviewing the manuscript. We have now referenced relevant Canadian evidence in the Introduction section:

'Two studies investigated patterns of mortality in adults with intellectual and developmental disabilities from Ontario, Canada. One of them reported a higher all-cause (6.1% vs. 1.6%) and amenable (21.4% vs. 14.1%) mortality levels compared with general population, but rates for all avoidable mortality were not provided.[12] The other study reported 1-year age-standardised mortality rates for years 2011-2014 to be between 30.3-37.4 for Manitoba adults with intellectual and developmental disabilities compared to the matched comparison group, meaning that 30.3-37.4 times more deaths occurred in this population than would be expected to occur in the Ontario population.[13] A further study on adults with intellectual and developmental disabilities from Manitoba, Canada reported crude avoidable premature mortality rates per 1,000 person-years to be between 2.3–3.3 for years 2013-2015, meaning that avoidable premature mortality was 2.3–3.3 times more prevalent among Manitoba adults with intellectual and developmental disabilities compared to the matched comparison group.[14]
